# Consistent Association of Type 2 Diabetes Risk Variants Found in Europeans in Diverse Racial and Ethnic Groups

**DOI:** 10.1371/journal.pgen.1001078

**Published:** 2010-08-26

**Authors:** Kevin M. Waters, Daniel O. Stram, Mohamed T. Hassanein, Loïc Le Marchand, Lynne R. Wilkens, Gertraud Maskarinec, Kristine R. Monroe, Laurence N. Kolonel, David Altshuler, Brian E. Henderson, Christopher A. Haiman

**Affiliations:** 1 Department of Preventive Medicine, Keck School of Medicine, University of Southern California/Norris Comprehensive Cancer Center, Los Angeles, California, United States of America; 2 Epidemiology Program, Cancer Research Center, University of Hawaii, Honolulu, Hawaii, United States of America; 3 Program in Medical and Population Genetics, Broad Institute of Massachusetts Institute of Technology and Harvard, Cambridge, Massachusetts, United States of America; 4 Center for Human Genetic Research, Massachusetts General Hospital, Boston, Massachusetts, United States of America; 5 Department of Molecular Biology, Massachusetts General Hospital, Boston, Massachusetts, United States of America; 6 Department of Genetics, Harvard Medical School, Boston, Massachusetts, United States of America; 7 Diabetes Unit, Massachusetts General Hospital, Boston, Massachusetts, United States of America; University of Oxford, United Kingdom

## Abstract

It has been recently hypothesized that many of the signals detected in genome-wide association studies (GWAS) to T2D and other diseases, despite being observed to common variants, might in fact result from causal mutations that are rare. One prediction of this hypothesis is that the allelic associations should be population-specific, as the causal mutations arose after the migrations that established different populations around the world. We selected 19 common variants found to be reproducibly associated to T2D risk in European populations and studied them in a large multiethnic case-control study (6,142 cases and 7,403 controls) among men and women from 5 racial/ethnic groups (European Americans, African Americans, Latinos, Japanese Americans, and Native Hawaiians). In analysis pooled across ethnic groups, the allelic associations were in the same direction as the original report for all 19 variants, and 14 of the 19 were significantly associated with risk. In summing the number of risk alleles for each individual, the per-allele associations were highly statistically significant (P<10^−4^) and similar in all populations (odds ratios 1.09–1.12) except in Japanese Americans the estimated effect per allele was larger than in the other populations (1.20; P_het_ = 3.8×10^−4^). We did not observe ethnic differences in the distribution of risk that would explain the increased prevalence of type 2 diabetes in these groups as compared to European Americans. The consistency of allelic associations in diverse racial/ethnic groups is not predicted under the hypothesis of Goldstein regarding “synthetic associations” of rare mutations in T2D.

## Author Summary

Single rare causal alleles and/or collections of multiple rare alleles have been suggested to create “synthetic associations” with common variants in genome-wide association studies (GWAS). This model predicts that associations with common variants will not be consistent across populations. In this study, we examined 19 T2D variants for association with T2D risk in 6,142 cases and 7,403 controls from five racial/ethnic populations in the Multiethnic Cohort (European Americans, African Americans, Latinos, Japanese Americans, and Native Hawaiians). In racial/ethnic pooled analysis, all 19 variants were associated with T2D risk in the same direction as previous reports in Europeans, and the sum total of risk variants was significantly associated with T2D risk in each racial/ethnic group. The consistent associations across populations do not support the Goldstein hypothesis that rare causal alleles underlie GWAS signals. We also did not find evidence that these markers underlie racial/ethnic disparities in T2D prevalence. Large-scale GWAS and sequencing studies in these populations are necessary in order to both improve the current set of markers at these risk loci and identify new risk variants for T2D that may be difficult, or impossible, to detect in European populations.

## Introduction

Multiple common risk alleles have been identified as reproducibly associated with risk of type 2 diabetes (T2D) [1]–[13]. With the exception of the *KCNQ1* locus which was identified in the Japanese population [1], [2], all of the well-replicated risk variants were first identified in populations of Northern European ancestry [3]–[13]. T2D morbidity varies widely across racial/ethnic groups; the prevalence is more than twice as high among African Americans, Japanese Americans, Latinos and Native Hawaiians as European Americans [14], [15]. It is important to evaluate whether and how genetic variation may contribute to health disparities between populations. For example, genetic variation at 8q24 may contribute to population differences in risk of prostate cancer [16], [17], and genetic variation at MYH9 contributes substantially to the higher rates of kidney disease in African Americans [18].

It has recently been argued that single rare causal variants and/or collections of multiple different rare variants on unrelated haplotypes may create “synthetic associations” of common variants with disease risk [19]–[21]. One prediction of this model is that the associations with common variants will not be consistent across populations (since many of the mutations will be young in age, and post-date the migrations that led to the founding of modern continental populations). Type 2 diabetes has been specifically discussed as a possible case in which synthetic associations might be operative, based on the lack of statistical significance in very small studies that examined allelic associations for T2D in multi-ethnic samples.

Testing the association of each validated risk allele for T2D in multiple populations is an important step to determine (a) whether these genetic markers can be used to better understand population risk in non-European populations, (b) to measure their association with racial/ethnic variation in disease risk, and (c) to test a prediction of the Goldstein “common SNP, rare mutation” hypothesis [19]–[21].

To allow for comparability of estimates of genetic risk among racial/ethnic groups requires large studies comprised of cases and controls defined using identical criteria and sampled ideally from the same study population. In the present study, we, as part of the Population Architecture using Genomics and Epidemiology (PAGE) Study, examined genetic associations with 19 validated risk alleles for T2D in European American, African American, Latino, Japanese American, and Native Hawaiian T2D cases (n = 6,142) and controls (n = 7,403) from the population-based Multiethnic Cohort study (MEC). We also evaluated whether these variants can be utilized to model the genetic risk of T2D in each population and their association to disparities in risk.

## Results

The age of the cases and controls ranged from 45 to 77 at cohort entry, with the mean age of cases (mean 59.0 years) being essentially the same as the controls (mean 58.8 years), and African Americans being on average the oldest (mean 60.2 years) and Native Hawaiians the youngest (mean 55.6 years). Compared to controls, cases were heavier, more likely to be a current or former smoker, less physically active and had fewer years of education (Table 1). Compared to the other groups, the Japanese were leaner (for cases and controls, men and women).

**Table 1:** The descriptive characteristics of type 2 diabetes cases and controls in the MEC at baseline by racial/ethnic group and sex.

Characteristic	European Americans		African Americans		Latinos		Japanese Americans		Native Hawaiians	
	Cases	Controls	Cases	Controls	Cases	Controls	Cases	Controls	Cases	Controls
**Men**										
N	288	504	408	634	1,063	1,051	978	986	258	493
Age (yr, mean ± SD)	57.6±8.1	57.5±8.0	62.6±7.9	61.9±7.9	59.7±6.9	59.7±6.9	59.0±8.2	59.1±8.2	55.5±7.0	55.6±7.0
BMI (kg/m^2^, mean ± SD)	29.4±4.7	25.1±3.1	28.9±4.5	26.2±3.8	28.4±4.2	26.2±3.3	26.9±4.0	24.5±3.1	31.0±4.8	28.0±5.2
Weight (lbs, mean ± SD)	209±38	180±25	206±37	187±30	188±31	174±25	172±30	156±23	215±38	194±40
Former/current smoker (%)	77.7	60.1	75.7	73.5	72.4	63.3	72.3	67.8	70.9	66.1
Education (≤12 years, %)	27.9	11.1	39.6	39.7	59.3	55.9	29.1	26.0	48.8	40.6
Physical activity (0 hrs / wk, %)^a^	30.9	21.0	45.8	38.3	34.6	29.5	35.3	30.9	24.4	19.3
**Women**										
N	245	502	669	835	1,157	1,133	758	775	318	490
Age (yr, mean ± SD)	58.1±8.3	58.3±8.3	59.1±8.6	58.6±8.6	59.0±7.1	59.1±7.0	59.4±8.5	59.5±8.4	55.7±7.6	55.7±7.6
BMI (kg/m^2^, mean ± SD)	30.2±6.2	24.4±4.5	31.8±6.3	27.6±5.7	30.0±5.9	26.2±4.4	26.6±4.6	22.8±3.5	31.4±6.7	26.4±5.2
Weight (lbs, mean ± SD)	179±39	147±28	191±39	166±35	170±34	148±26	145±27	124±21	186±42	155±33
Former or current smoker (%)	45.7	48.6	51.9	51.3	36.3	31.0	35.6	30.2	51.3	47.1
Education (≤12 years, %)	30.2	20.9	37.1	30.5	74.9	68.5	34.7	31.9	51.4	46.0
Physical activity (0 hrs / wk, %)^a^	55.1	44.2	64.6	58.6	66.8	59.7	64.3	61.9	50.3	37.1

a Hours per week of vigorous activity of both work and sports.

**Figure 1:**
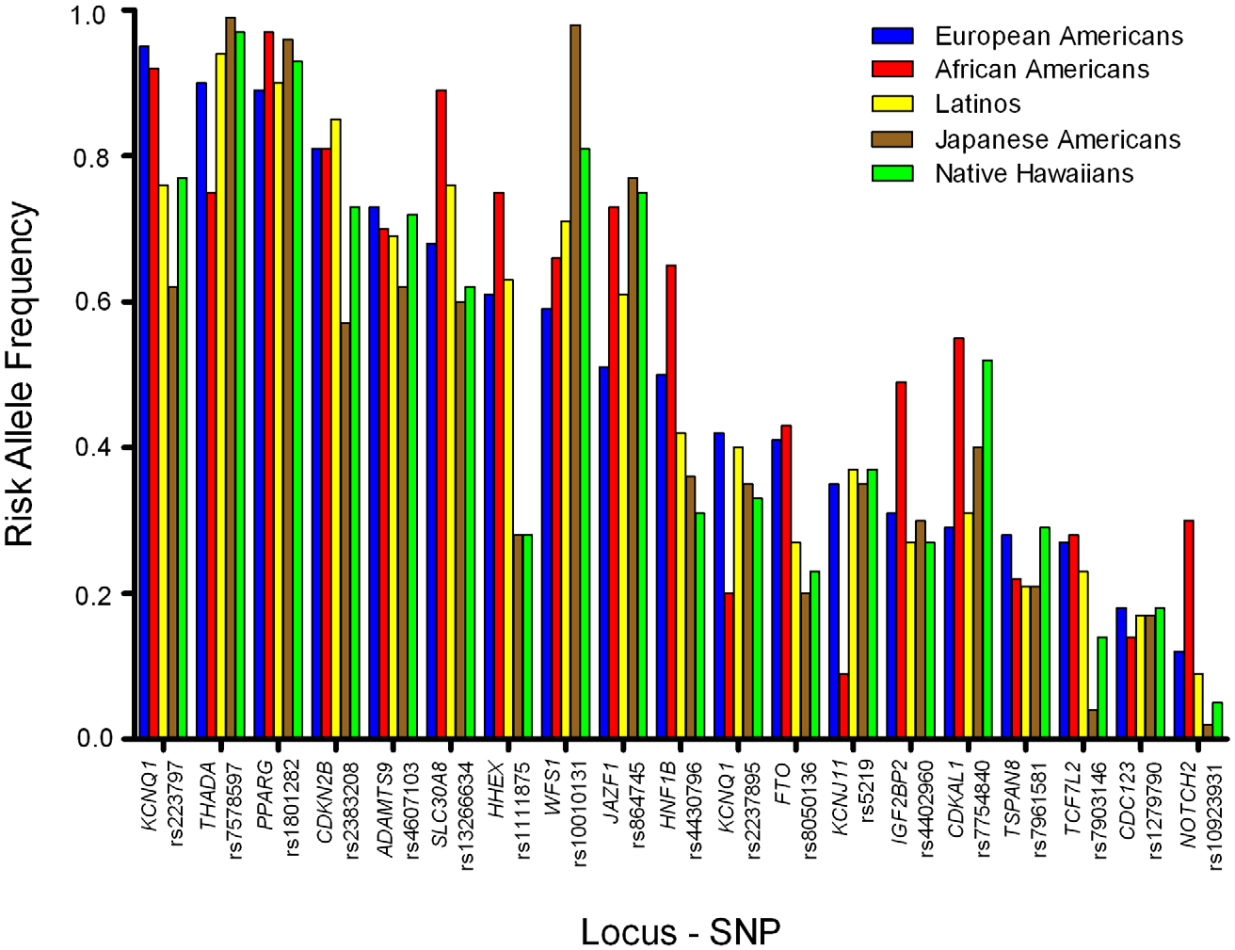
Risk allele frequencies by racial/ethnic group. Risk allele frequencies for each variant in European Americans (Blue), African Americans (Yellow), Latinos (Purple), Japanese Americans (Red), and Native Hawaiians (Green). The order of the variants is based on the frequency of the risk allele in European Americans (high to low).

The established T2D risk SNPs were polymorphic (frequency>0.05) in all racial/ethnic groups (Figure 1), except for rs10923931 (*NOTCH2*) in Japanese and Native Hawaiians and rs7903146 (*TCF7L2*) in Japanese (Table 2). In European populations these 19 SNPs have very modest odds ratios (1.1–1.3 per copy of the risk allele), and required studies of more than ten thousand cases and controls to reach genome-wide significance [3]–[13], [22]. Our sample sizes, although substantial, provided limited power to detect these modest effects (Table S1; power to achieve nominal significance (P = 0.05) of 34%, 47%, 67%, 54%, and 33%, in European Americans, African Americans, Latinos, Japanese Americans, and Native Hawaiians, respectively).

**Table 2:** The association of known risk alleles for T2D by race/ethnicity.^a^ ^b^

SNP	Risk Allele^c^	Chr.	NearestGene		European Americans533 cases1,006 controls	African Americans1,077 cases1,469 controls	Latinos2,220 cases2,184 controls	Japanese Americans1,736 cases1,761 controls	Native Hawaiians576 cases983 controls	Pooled6,142 cases7,403 controls	P value	P_het_ d
rs10923931	T	1	*NOTCH2*	OR(95%CI)	0.84 (0.64–1.10)	1.10 (0.97–1.25)	1.17 (1.01–1.35)	1.00 (0.70–1.44)	0.76 (0.50–1.15)	1.06 (0.98–1.16)	0.16	0.086
				RAF	0.12	0.29	0.09	0.02	0.05	0.11		
rs7578597	T	2	*THADA*	OR(95%CI)	1.42 (1.06–1.91)	1.04 (0.90–1.19)	1.12 (0.93–1.35)	1.10 (0.68–1.78)	1.65 (1.01–2.70)	1.13 (1.02–1.25)	0.016	0.21
				RAF	0.90	0.75	0.94	0.99	0.97	0.91		
rs1801282	C	3	*PPARG*	OR(95%CI)	1.26 (0.95–1.68)	1.95 (1.30–2.92)	1.03 (0.89–1.19)	1.05 (0.82–1.36)	1.14 (0.84–1.55)	1.13 (1.02–1.26)	0.018	0.048
				RAF	0. 89	0.97	0.90	0.96	0.93	0.93		
rs4607103	C	3	*ADAMTS9*	OR(95%CI)	1.11 (0.92–1.34)	1.03 (0.91–1.18)	0.99 (0.89–1.08)	1.05 (0.94–1.17)	0.98 (0.82–1.16)	1.02 (0.97–1.08)	0.45	0.78
				RAF	0.73	0.70	0.69	0.61	0.72	0.68		
rs4402960	T	3	*IGF2BP2*	OR(95%CI)	1.02 (0.85–1.23)	1.14 (1.01–1.28)	1.05 (0.95–1.16)	1.24 (1.11–1.38)	1.17 (0.99–1.39)	1.13 (1.07–1.19)	2.2×10^−5^	0.26
				RAF	0.31	0.49	0.27	0.30	0.27	0.33		
rs10010131	G	4	*WFS1*	OR(95%CI)	1.18 (0.99–1.40)	0.94 (0.83–1.07)	1.15 (1.04–1.27)	1.45 (0.98–2.13)	1.27 (1.03–1.56)	1.11 (1.04–1.19)	1.8×10^−3^	0.032
				RAF	0.59	0.66	0.71	0.98	0. 81	0.76		
rs7754840	C	6	*CDKAL1*	OR(95%CI)	1.26 (1.05–1.50)	1.03 (0.92–1.16)	1.09 (0.99–1.19)	1.37 (1.24–1.52)	1.39 (1.19–1.62)	1.20 (1.13–1.26)	4.1×10^−11^	6.2×10^−4^
				RAF	0.29	0.55	0.31	0.40	0.52	0.40		
rs864745	T	7	*JAZF1*	OR(95%CI)	0.98 (0.83–1.16)	1.16 (1.01–1.32)	1.30 (1.19–1.43)	1.19 (1.05–1.35)	1.13 (0.93–1.36)	1.20 (1.13–1.27)	1.3×10^−9^	0.054
				RAF	0.51	0.73	0.61	0.77	0.75	0.68		
rs13266634	C	8	*SLC30A8*	OR(95%CI)	1.28 (1.06–1.54)	1.21 (0.99–1.48)	1.11 (1.00–1.23)	1.18 (1.06–1.31)	1.04 (0.88–1.22)	1.15 (1.08–1.22)	9.7×10^−6^	0.47
				RAF	0.68	0. 89	0.75	0.60	0.62	0.72		
rs2383208	A	9	*CDKN2B*	OR(95%CI)	1.35 (1.07–1.69)	1.14 (0.98–1.33)	1.15 (1.01–1.31)	1.26 (1.13–1.40)	1.02 (0.85–1.22)	1.18 (1.11–1.26)	2.1×10^−7^	0.27
				RAF	0. 81	0. 81	0. 85	0.56	0.74	0.75		
rs1111875	C	10	*HHEX*	OR(95%CI)	0.93 (0.78–1.11)	1.10 (0.95–1.26)	1.03 (0.94–1.13)	1.21 (1.08–1.36)	0.93 (0.78–1.11)	1.07 (1.01–1.13)	0.028	0.037
				RAF	0.61	0.74	0.63	0.28	0.28	0.52		
rs7903146	T	10	*TCF7L2*	OR(95%CI)	1.55 (1.29–1.87)	1.32 (1.16–1.51)	1.31 (1.19–1.45)	1.74 (1.38–2.20)	1.12 (0.90–1.40)	1.36 (1.27–1.45)	1.1×10^−19^	0.068
				RAF	0.27	0.28	0.23	0.04	0.14	0.19		
rs12779790	G	10	*CDC123*	OR(95%CI)	1.02 (0.82–1.28)	1.09 (0.92–1.29)	1.19 (1.06–1.33)	1.01 (0.88–1.15)	1.16 (0.95–1.41)	1.10 (1.03–1.18)	5.9×10^−3^	0.36
				RAF	0.17	0.14	0.17	0.17	0.18	0.17		
rs2237895^e^	C	11	*KCNQ1*	OR(95%CI)	0.98 (0.82–1.16)	1.04 (0.90–1.21)	1.15 (1.05–1.28)	1.12 (0.98–1.27)	1.16 (0.97–1.39)	1.11 (1.04–1.18)	7.8×10^−4^	0.17
				RAF	0.42	0.20	0.40	0.35	0.33	0.34		
rs2237897^e^	C	11	*KCNQ1*	OR(95%CI)	0.86 (0.59–1.26)	1.13 (0.90–1.42)	1.23 (1.09–1.39)	1.26 (1.11–1.44)	1.06 (0.86–1.31)	1.21 (1.13–1.30)	3.2×10^−7^	0.10
				RAF	0.95	0.92	0.76	0.62	0.78	0.79		
rs5219	T	11	*KCNJ11*	OR(95%CI)	1.24 (1.05–1.47)	1.03 (0.84–1.26)	1.09 (1.00–1.20)	1.26 (1.13–1.40)	1.03 (0.88–1.21)	1.15 (1.08–1.21)	3.3×10^−6^	0.13
				RAF	0.35	0.09	0.37	0.35	0.37	0.31		
rs7961581	C	12	*TSPAN8*	OR(95%CI)	1.03 (0.86–1.24)	0.92 (0.80–1.06)	1.03 (0.93–1.15)	1.00 (0.88–1.14)	1.12 (0.94–1.33)	1.01 (0.95–1.08)	0.71	0.51
				RAF	0.29	0.23	0.21	0.21	0.29	0.23		
rs8050136	A	16	*FTO*	OR(95%CI)	0.89 (0.75–1.06)	1.07 (0.95–1.20)	1.02 (0.92–1.12)	1.04 (0.92–1.18)	1.01 (0.84–1.21)	1.02 (0.96–1.08)	0.48	0.68
				RAF	0.41	0.43	0.27	0.20	0.23	0.30		
rs4430796	G	17	*HNF1B*	OR(95%CI)	0.96 (0.82–1.14)	1.10 (0.97–1.25)	0.96 (0.88–1.05)	1.18 (1.06–1.32)	1.09 (0.92–1.30)	1.05 (1.00–1.11)	0.058	0.043
				RAF	0.50	0.65	0.42	0.36	0.31	0.45		

a Odds ratios (and 95% confidence intervals) for allele dosage effects are adjusted for age (quartiles), BMI (quartiles), sex, and ethnicity (in pooled analysis).

b Ethnic specific risk allele frequencies (RAF) calculated for each SNP.

c NCBI build 36 (forward strand).

d P_het_ = P value for heterogeneity of allelic effects across ethnic groups (4 df test).

e rs2237895 and rs2237897 adjusted for each other.

We first assessed whether the “risk allele” of each SNP was associated in the same direction (odds ratios>1) in each ethnic group. Whereas the null hypothesis is that 50% of “risk” alleles would trend in the same direction, we observed from 12 (63%; P = 0.18; binomial probability) in European Americans to 19 (100%; P = 1.9×10^−6^) in Japanese Americans. The number of these associations that reached nominal significance (P<0.05) ranged from 3 (P = 0.067; binomial probability) in Native Hawaiians to 10 (P = 5.9×10^−9^) in Japanese (Table 2). For the majority of alleles with positive associations, odds ratios for homozygous carriers were greater than for heterozygous carriers in each population, which provides support for their associations and allele dosage effects (Table S2). In African Americans, results were similar after adjustment for percent European ancestry (Table S3). Adjustment for education, a proxy for socio-economic status (SES) and European ancestry, did not influence the results (Table S4) [23].

We next performed analyses that combined evidence for association across the five ethnic groups. In this analysis the power to achieve nominal significance for the allelic effects reported previously was >80% for 18 out of 19 alleles (average 94%; Table S1). In this analysis all 19 (100%; P = 1.9×10^−6^, binomial probability) variants were associated with risk in the same direction as the initial report (odds ratios>1) and 14 (P = 5.7×10^−15^; binomial probability) with nominal statistical significance (P<0.05). All 19 associations remained in the same direction as previous reports (OR>1) and 13 of the variants were significantly associated with T2D risk when the European American subjects were excluded from the analysis. The association of rs8050136 in *FTO* was attenuated by adjustment for BMI (odds ratio (95% confidence interval), 1.06(1.00–1.11) prior to adjustment; 1.02(0.96–1.08) after adjustment). Only 5 of the 19 risk variants showed nominal evidence for heterogeneity in the odds ratio across ethnic groups, and only one of these (*CDKAL1*) was significant after correction for having performed 19 tests (*PPARG*, rs1801282, P_het_ = 0.048; *WFS1*, rs10010131, P_het_ = 0.032; *CDKAL1*, rs7754840, P_het_ = 6.2×10^−4^; *HHEX*, rs1111875, P_het_ = 0.037; and, *HNF1B*, rs4430796, P_het_ = 0.043; Table 2).

### Summary Measures of Risk

We next calculated a summary risk score comprised of an unweighted count of the 19 risk-associated alleles. The average increment in risk per allele was generally similar in all populations, except Japanese Americans, where the effect of each allele was nearly double that observed in Europeans ((odds ratio, 95% confidence interval): African Americans, 1.09, 1.05–1.12; (P = 3.0×10^−6^); Native Hawaiians, 1.10, 1.06–1.15 (P = 1.2×10^−5^); European Americans, 1.11, 1.06–1.17 (P = 1.2×10^−5^); Latinos, 1.12, 1.09–1.14 (P = 7.5×10^−19^); and, Japanese, 1.20, 1.17–1.24; (P = 7.0×10^−32^); P_het_ = 3.8×10^−4^). Results were similar when limiting the analysis to individuals with complete genotype data for all variants and when including only those markers associated with risk (at P<0.10) (Table S5). Individuals in the top quartile of the risk allele distribution were at 1.6 (African Americans, P = 5.3×10^−4^) to 3.1-fold (Japanese Americans, P = 7.9×10^−26^) greater risk of diabetes compared to those in the lowest quartile (Table 3).

**Table 3:** The association of the total risk score with T2D risk by racial/ethnic population.^a^

		European Americans	African Americans	Latinos	Japanese Americans	Native Hawaiians	Pooled
Total Risk alleles, Mean (range)		18.8 (10–27)	20.2 (11–27)	18.6 (10–28)	17.2 (10–26)	18.1 (10–26)	18.5 (10–28)
**Per allele additive model**							
n (cases/controls)		533/1,006	1,077/1,469	2,220/2,184	1,736/1,761	576/983	6,142/7,403
OR(95% CI)^b^ ^c^		1.11 (1.06–1.17)	1.09 (1.05–1.12)	1.12 (1.09–1.14)	1.20 (1.17–1.24)	1.10 (1.06–1.15)	1.13 (1.11–1.15)
P-value		1.2×10^−5^	3.0×10^−6^	7.5×10^−19^	7.0×10^−32^	1.2×10^−5^	4.7×10^−59^
P-value vs. European Americans		Ref.	0.43	0.76	0.007	0.86	
**Quartiles of Risk Alleles** d							
Q1	n (cases/controls)	69/196	206/360	362/464	267/441	119/251	1,023/1,712
	OR(95% CI)	1.00(ref)	1.00(ref)	1.00(ref)	1.00(ref)	1.00(ref)	1.00(ref)
Q2	n (cases/controls)	112/273	273/437	520/605	424/518	150/307	1,479/2,140
	OR(95% CI)	1.17 (0.79–1.73)	1.10 (0.86–1.40)	1.17 (0.97–1.42)	1.39 (1.12–1.72)	1.11 (0.81–1.52)	1.21 (1.08–1.35)
	P-value	0.44	0.45	0.10	3.2×10^−3^	0.51	8.4×10^−4^
Q3	n (cases/controls)	170/281	353/391	626/587	297/306	167/259	1,613/1,824
	OR(95% CI)	1.52 (1.05–2.21)	1.59 (1.25–2.01)	1.50 (1.25–1.81)	1.85 (1.46–2.36)	1.39 (1.02–1.90)	1.61 (1.44–1.80)
	P-value	0.027	1.3×10^−4^	2.1×10^−5^	5.4×10^−7^	0.039	4..2×10^−17^
Q4	n (cases/controls)	182/256	245/281	712/528	748/496	140/166	2,027/1,727
	OR(95% CI)	1.88 (1.29–2.74)	1.58 (1.22–2.04)	1.99 (1.65–2.40)	3.06 (2.48–3.77)	2.09 (1.49–2.94)	2.17 (1.95–2.42)
	P-value	9.3×10^−4^	5.3×10^−4^	7.6×10^−13^	7.9×10^−26^	2.0×10^−5^	4.6×10^−44^

a Odds ratios (and 95% confidence intervals) adjusted for age (quartiles), BMI (quartiles), sex, and ethnicity (in pooled analysis).

b P = 3.8×10^−4^ for heterogeneity of allelic effects across ethnic groups (4 df test).

c P≤0.02 for each ethnic group for the comparison with Japanese Americans.

d Quartiles are defined separately for each population.

**Figure 2:**
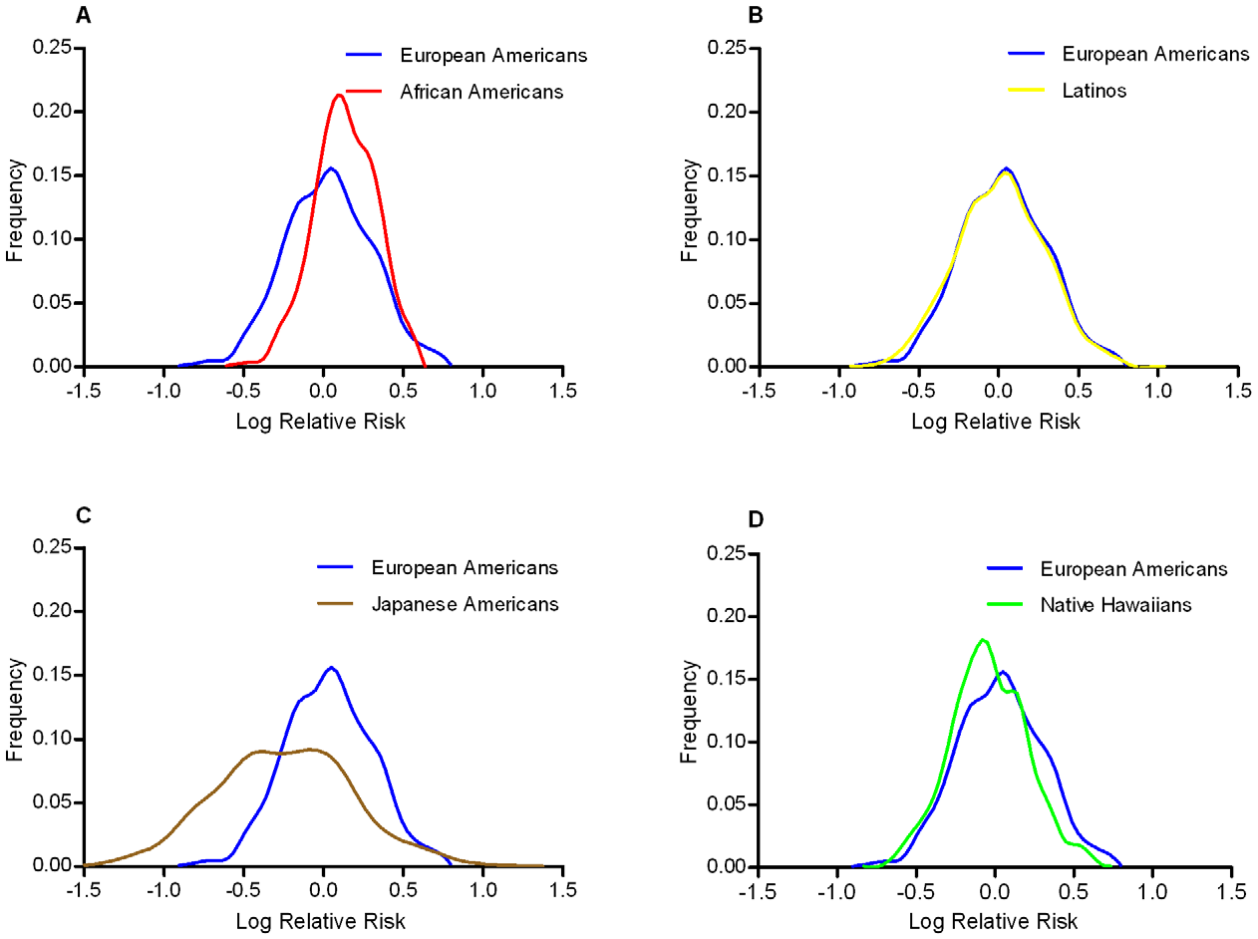
Predicted distribution of T2D risk from common variants by racial/ethnic group compared to European Americans. Comparison of the predicted risk distributions conveyed by the risk alleles relative to European Americans (Blue in Panels A–D): Panel A, African Americans (Red); Panel B, Latinos (Yellow); Panel C, Japanese Americans (Brown); Panel D, Native Hawaiians (Green). The x-axis is the log relative risk for each population centered (at log RR = 0) around the average total risk allele count in the multiethnic sample (18.5). The y-axis is the relative frequency of the population with that level of risk.

Using these ethnic-specific per allele odds ratio estimates and the aggregate risk allele counts, we built a quantitative risk model to compare the distribution of genetic risks between populations associated with these marker alleles. The higher average number of risk alleles in African Americans caused their distribution to be slightly right shifted (towards higher log ORs) compared to European Americans, however their relatively smaller per allele odds ratio resulted in wide overlap with the European American distribution (Figure 2). The Japanese Americans had a wider distribution of risk because of the large per allele odds ratio, but the low average risk allele counts caused the Japanese distribution to be left-shifted (towards lower log ORs) compared to European Americans. The distributions for Latinos and Native Hawaiians were very similar to the European Americans.

## Discussion

We tested 19 common genetic risk markers that were discovered in European populations. We found that association with all 19 of these SNPs trended in the same direction in this large multiethnic study, and the majority of these variants were nominally significant in their association with diabetes risk. A risk score comprised of these alleles was significantly associated with diabetes risk in all five racial/ethnic groups, with the only significant heterogeneity being larger effect sizes in Japanese Americans. However, in comparing the distribution of risk conferred by these alleles between populations we found that they explain little, if any, of known differences in the prevalence of diabetes between these populations.

These observations indicate that most, if not all, of these alleles show directionally similar association to T2D across many populations. Such a pattern indicates that the causal alleles at these validated risk loci (which have yet to be found) likely predate the migrations that separated these populations now residing in Europe, Africa, East Asia, the Pacific Islands and the Americas. We note that this pattern is unexpected under the recently described “common SNP, rare mutation” model of Goldstein that suggests that GWAS signals with common alleles for T2D and other diseases may be “synthetic associations” created by one or more rare alleles [19]–[21]. Under the Goldstein Hypothesis the consistent associations that we noted at these loci across populations would only be observed if, in each population, one or more distinct rare alleles arose at each locus, and they happened to arise each time on the same haplotype background. Although possible, this scenario seems unlikely, and a more parsimonious explanation would be the “synthetic association” hypothesis of Goldstein does not apply to a majority of these T2D SNPs.

The modest number of cases and controls in this study (as compared to the initial discovery studies) likely underlies the lack of statistically significant associations in some groups. Weaker associations in some racial/ethnic groups may also be due to differences in allele frequencies, linkage disequilibrium, and environmental and genetic modifiers. In two cases (*WFS1* and *CDKAL1*), significant heterogeneity by race/ethnicity reflected a lack of association in African Americans, perhaps because of lower linkage disequilibrium between the marker and the biologically relevant allele.

It is interesting that the odds ratios observed for these marker SNPs were larger in Japanese Americans than in the original discovery cohorts, and in the other ethnic groups in our study. A meta-analysis of 7 association studies in Japanese populations replicated associations from studies in European populations for 7 loci under study (*TCF7L2*, *CDKAL1*, *CDKN2B*, *IGF2BP2*, *SLC30A8*, *KCNJ11*, and *HHEX*) [24]. A recent GWAS in Japanese observed significant associations in *KCNQ1* as well as these same 7 loci and, similar to our observations, noted magnitudes of effect that were generally stronger than previously observed in European populations [25]. Additional studies in other Asian populations have replicated associations with many of these loci as well [24], [26]–[28].

In the Multiethnic Cohort, we have found the prevalence of T2D to be at least 2-fold higher in African Americans, Latinos, Japanese and Native Hawaiians compared to European Americans, with these differences being independent of body weight [14]. We examined the extent to which the known genetic risk alleles for diabetes could explain these disparities by quantifying and comparing the relative risk distributions between populations. Compared to European Americans, we did not observe evidence of greater genetic risk in any population. Our findings therefore indicate that these risk markers explain little, if any, of racial/ethnic disparities in T2D prevalence. It remains possible that the actual causal alleles in these regions may be more common in frequency and/or have larger effects than the index signals in non-European populations. As seen with *KCNQ1* [1], [2], GWAS in non-European populations are effective in discovering risk loci that are important in multiple populations but difficult to identify in European populations where the alleles are rare.

This study had a number of limitations. First, a self-report of diabetes and use of medication for diabetes was used to define cases and controls. We observed that approximately 1% of a random sample of the controls in this study had HbA1C levels above 7.0%, which suggests that only a small portion of controls had undiagnosed diabetes (see Materials and Methods). Also, our case definition did not differentiate between T1D and T2D, however we expect this misclassification to be minor as <3% of T2D cases had a previous diagnosis of T1D based on other sources (see Materials and Methods). The highly consistent findings of this study, as compared to the discovery GWAS reports, argue that our phenotypic characterization is adequate to observe the association to T2D.

Some caution should also be given to the interpretation of the risk modeling conducted in each ethnic group, as the genetic markers included are unlikely to be the causal alleles. Future fine-mapping and sequencing studies to identify the functional variants (common and/or rare) and large-scale testing of each allele will be required to more precisely model risk as well as assess differences in the distribution of genetic risk across populations.

Another limitation is that we did not account for the potential confounding effects of population stratification. However, odds ratios were essentially unchanged after adjusting for global European ancestry in a subset of African Americans (336 cases 397 controls) for whom ancestry markers were available, suggesting that effects due to population substructure were not substantial, at least in this group. We also noted that controlling for education, a proxy for SES which has been shown to be significantly associated with Native American ancestry in Latinos [23], had little effect on the associations with these risk alleles. Furthermore, the risk alleles were not generally more frequent in Latinos than in European Americans which would be likely if these alleles were proxies for more general ancestry differences. While population stratification is unlikely to fully explain these findings, it remains possible that at some loci, the causal alleles may be more correlated with ancestry than the index SNPs.

In summary, our data provide strong support for common genetic variation contributing to T2D risk in multiple populations. Our findings in T2D do not support the theory that GWAS signals are due to rare alleles. Nonetheless, GWAS and sequencing studies in these and other racial/ethnic populations are needed to reveal a more complete spectrum of risk alleles that are important globally as well as those that may contribute to risk disparities.

## Materials and Methods

### Ethics Statement

The Institutional Review Boards at the University of Southern California and University of Hawaii approved the study protocol.

### Study Population

The MEC consists of 215,251 men and women, and comprises mainly five self-reported racial/ethnic populations: European Americans, African Americans, Latinos, Japanese Americans and Native Hawaiians [29]. Between 1993 and 1996, adults between 45 and 75 years old were enrolled by completing a 26-page, self-administered questionnaire asking detailed information about dietary habits, demographic factors, level of education, personal behaviors, and history of prior medical conditions (e.g. diabetes). Potential cohort members were identified through Department of Motor Vehicles drivers' license files, voter registration files and Health Care Financing Administration data files. In 2001, a short follow-up questionnaire was sent to update information on dietary habits, as well as to obtain information about new diagnoses of medical conditions since recruitment. Between 2003 and 2007, we re-administered a modified version of the baseline questionnaire. All questionnaires inquired about history of diabetes, without specification as to type (1 vs. 2). Between 1995 and 2004, blood specimens were collected from ∼67,000 MEC participants at which time a short questionnaire was administered to update certain exposures, and collect current information about medication use.

Cohort members in California are linked each year to the California Office of Statewide Health Planning and Development (OSHPD) hospitalization discharge database which consists of mandatory records of all in-patient hospitalizations at most acute-care facilities in California. Records include information on the principal diagnosis plus up to 24 other diagnoses (coded according to ICD-9), including T1D and T2D. In Hawaii cohort members have been linked with the diabetes care registries for subjects with Hawaii Medical Service Association (HMSA) and Kaiser Permanente Hawaii (KPH) health plans (∼90% of the Hawaiian population has one of these two plans) [15]. Information from these additional databases have been utilized to assess the percentage of T2D controls (as defined below) with undiagnosed T2D, as well as the percentage of identified diabetes cases with T1D rather than T2D. Based on the OSHPD database <3% of T2D cases had a previous diagnosis of T1D. We did not use these sources to identify T2D cases because they did not include information on diabetes medications, one of our inclusion criteria for cases (see below).

In this study, diabetic cases were defined using the following criteria: (a) a self-report of diabetes on the baseline questionnaire, 2^nd^ questionnaire or 3^rd^ questionnaire; and (b) self-report of taking medication for T2D at the time of blood draw; and (c) no diagnosis of T1D in the absence of a T2D diagnosis from the OSHPD (California Residents). Controls were defined as: (a) no self-report of diabetes on any of the questionnaires while having completed a minimum of 2 of the 3 (79% of controls returned all 3 questionnaires); and (b) no use of medications for T2D at the time of blood draw; and (c) no diabetes diagnosis (type 1 or 2) from the OSHPD, HMSA or KPH registries. To preserve DNA for genetic studies of cancer in the MEC, subjects with an incident cancer diagnosis at time of selection for this study were excluded. Controls were frequency matched to cases on age at entry into the cohort (5-year age groups) and for Latinos, place of birth (U.S. vs. Mexico, South or Central America), oversampling African American, Native Hawaiian and European American controls to increase statistical power.

Fasting glucose (FG) and HbA_1C_ measurements were used to validate the case-control selection criteria. Among 185 T2D cases and 1,048 controls who met the T2D case-control definitions above and with FG measurements available from ongoing studies in the MEC, 57% of cases (ranging from 43% in European Americans to 63% in Japanese Americans) and 3% of controls (ranging from 1% in African Americans to 6% in Latinos) had a FG value >125 mg/dl. We also measured HbA_1C_ (ARUP Laboratories, Salt Lake City, Utah) in 50 cases and 50 controls per each sex-ethnic group. Just over 1% (6/500) of controls were likely to have unreported T2D (HbA_1C_ value ≥7%). In contrast, ∼47% (234/500) of T2D cases had HbA_1C_ ≥7% (ranging from 41% in European Americans to 57% of Native Hawaiians). Since hypoglycemic medication use was part of the case selection criteria, some cases were expected to have FG and HbA_1C_ levels in the normal range.

Altogether, this study included 6,142 T2D cases and 7,403 controls (European American (533/1,006), African American (1,077/1,469), Latino (2,220/2,184), Japanese American (1,736/1,761) and Native Hawaiian (576/983)). Genotyping was conducted by the TaqMan allelic discrimination assay (Applied Biosystems, Foster City, CA) [30]. For all SNPs, genotype call rates were >95% among case and control groups in each population and HWE p-values among controls were >0.05 in at least 4 of the 5 ethnic groups and none of the values were <0.01 (Table S6). Subjects missing data for >5 SNPs (n = 82) were removed from the analysis.

### Statistical Analysis

Odds ratios and 95% confidence intervals were calculated for each allele in unconditional logistic regression models while adjusting for age at cohort entry (quartiles), body mass index (BMI, kg/m^2^, quartiles), sex, and race/ethnicity (pooled analysis) in ethnic-stratified and pooled analyses. Associations with the two variants at *KCNQ1* were examined adjusting for the other allele. Potential confounding factors including, smoking history, education, physical activity, and history of hypertension were evaluated but did not influence the results. Potential confounding by percent European ancestry was examined in a subset of African American men (336 cases, 397 controls) with available genetic ancestry information [16], [31], [32].

We also modeled the cumulative genetic risk of T2D using these markers. We summed the number of risk alleles for each individual and estimated the odds ratio per allele for this aggregate unweighted allele count variable as an approximate risk score appropriate for unlinked variants with independent effects of approximately the same magnitude for each allele. We also examined a second model where each allele was weighted and multiplied by the log of the published odds ratio prior to summing all alleles. The results of the more parsimonious unweighted risk score is presented as the two risk scores were highly correlated in each ethnic group (Pearson r≥0.92) and similar associations with T2D risk were observed for each score. For individuals missing genotypes for a given SNP, we assigned the average number of risk alleles within each ethnic group (2× risk allele frequency) to replace the missing value for that SNP. We used these ethnic-specific per allele summary odds ratios and the total number of risk alleles among control subjects to estimate the distribution of relative risks conveyed by all risk alleles. To avoid making the reference group carriers of zero risk alleles (a group which does not exist) we centered the distribution on the mean number of risk alleles observed in the control population (18.5). The log relative risk for each subject was calculated as logRR = (RA−18.5)×log(OR_i_) (where RA is equal to the subject's total risk alleles and log(OR_i_) is the log of the ethnic specific per allele odds ratio. A spline function was used to capture the shape of the distributions of log OR for display purposes. Two variants in *KCNQ1* were included in the risk modeling because both were significantly associated with T2D when co-modeled (results were similar when only the most significant of the two, rs2237897, was included). The variant in FTO was excluded from risk modeling procedures, as we found (as have others) that it is not a risk factor for diabetes independent of its effect on obesity.

## Supporting Information

Table S1 Power estimates (α = 0.05) to detect relative risks in previous studies.
(0.10 MB DOC)

Table S2 Association with T2D risk by genotype.
(0.13 MB DOC)

Table S3 Effects of European ancestry adjustment in African Americans.
(0.07 MB DOC)

Table S4 Effects of adjustment for education.
(0.12 MB DOC)

Table S5 Associations with risk score among subjects with and without complete genotype data.
(0.05 MB DOC)

Table S6 Genotyping efficiency: genotype call rates, Hardy-Weinberg Equilibrium testing.
(0.08 MB DOC)
