# Supplementary material for: Consistent Association of Type 2 Diabetes Risk Variants Found in Europeans in Diverse Racial and Ethnic Groups
Source: PLoS Genet. 2010 Aug 26;6(8):e1001078. doi: 10.1371/journal.pgen.1001078 (PMC2928808; doi:10.1371/journal.pgen.1001078)
Supplement: Table S1 — Power estimates (α = 0.05) to detect relative risks in previous studies. (0.10 MB DOC) [file pgen.1001078.s001.doc]

**Table S1: Power estimates (α=0.05) to detect relative risks in previous studies.**

|  |  | European Americans  533 cases  1,006 controls | | African Americans  1,077 cases  1,469 controls | | Latinos  2,220 cases  2,184 controls | | Japanese Americans  1,736 cases  1,761 controls | | Native Hawaiians  576 cases  983 controls | | Pooled  6,142 cases  7,403 controls | |
| --- | --- | --- | --- | --- | --- | --- | --- | --- | --- | --- | --- | --- | --- |
| SNP | Published odds ratios^a^ | RAF | Estimated Power | RAF | Estimated Power | RAF | Estimated Power | RAF | Estimated Power | RAF | Estimated Power | RAF | Estimated Power |
| rs10923931 | 1.13 | 0.12 | 19% | 0.29 | 51% | 0.09 | 39% | 0.02 | 11% | 0.05 | 11% | 0.11 | 89% |
| rs7578597 | 1.15 | 0.90 | 19% | 0.75 | 55% | 0.94 | 33% | 0.99 | 9% | 0.97 | 10% | 0.91 | 89% |
| rs1801282 | 1.14 | 0.89 | 18% | 0.97 | 12% | 0.90 | 44% | 0.96 | 18% | 0.93 | 14% | 0.93 | 76% |
| rs4607103 | 1.09 | 0.73 | 17% | 0.70 | 28% | 0.69 | 46% | 0.61 | 42% | 0.72 | 18% | 0.68 | 90% |
| rs4402960 | 1.14 | 0.31 | 37% | 0.49 | 64% | 0.27 | 79% | 0.30 | 72% | 0.27 | 36% | 0.33 | 99% |
| rs10010131 | 1.11 | 0.59 | 27% | 0.66 | 41% | 0.71 | 59% | 0.98 | 9% | 0.81 | 19% | 0.76 | 95% |
| rs7754840 | 1.12 | 0.28 | 28% | 0.55 | 51% | 0.31 | 70% | 0.40 | 65% | 0.52 | 33% | 0.40 | 99% |
| rs864745 | 1.10 | 0.51 | 24% | 0.73 | 31% | 0.61 | 58% | 0.77 | 38% | 0.75 | 20% | 0.68 | 95% |
| rs13266634 | 1.12 | 0.68 | 28% | 0.89 | 23% | 0.75 | 62% | 0.60 | 64% | 0.62 | 31% | 0.72 | 98% |
| rs2383208 | 1.20 | 0.81 | 45% | 0.81 | 69% | 0.85 | 84% | 0.56 | 96% | 0.74 | 56% | 0.75 | 99% |
| rs1111875 | 1.13 | 0.61 | 35% | 0.74 | 46% | 0.63 | 78% | 0.28 | 64% | 0.28 | 32% | 0.52 | 99% |
| rs7903146 | 1.37 | 0.27 | 97% | 0.28 | 99% | 0.23 | 99% | 0.04 | 79% | 0.14 | 88% | 0.19 | 99% |
| rs12779790 | 1.11 | 0.17 | 18% | 0.14 | 26% | 0.17 | 46% | 0.17 | 38% | 0.18 | 20% | 0.17 | 90% |
| rs2237895 | 1.23 | 0.42 | 77% | 0.20 | 85% | 0.40 | 99% | 0.35 | 99% | 0.33 | 76% | 0.34 | 99% |
| rs2237897^b^ | 1.19 | 0.95 | 16% | 0.92 | 36% | 0.76 | 93% | 0.62 | 94% | 0.78 | 47% | 0.79 | 99% |
| rs5219 | 1.14 | 0.35 | 38% | 0.09 | 27% | 0.37 | 85% | 0.35 | 75% | 0.37 | 40% | 0.31 | 99% |
| rs7961581 | 1.09 | 0.28 | 18% | 0.23 | 25% | 0.21 | 39% | 0.21 | 32% | 0.29 | 19% | 0.23 | 85% |
| rs8050136 | 1.17 | 0.41 | 54% | 0.43 | 79% | 0.27 | 91% | 0.20 | 77% | 0.23 | 44% | 0.30 | 99% |
| rs4430796 | 1.10 | 0.50 | 24% | 0.65 | 36% | 0.42 | 60% | 0.36 | 49% | 0.31 | 22% | 0.45 | 97% |

^a^Odds ratio estimates from previous scans in European populations.[4,5,7,12,13,22]

^b^Odds ratio estimate for rs2237897 from personal communication (Ben Voight, DIAGRAM consortium, v1)
