# Supplementary material for: Consistent Association of Type 2 Diabetes Risk Variants Found in Europeans in Diverse Racial and Ethnic Groups
Source: PLoS Genet. 2010 Aug 26;6(8):e1001078. doi: 10.1371/journal.pgen.1001078 (PMC2928808; doi:10.1371/journal.pgen.1001078)
Supplement: Table S2 — Association with T2D risk by genotype. (0.13 MB DOC) [file pgen.1001078.s002.doc]

**Table S2: Association with T2D risk by genotype.a**

| SNP | Risk^b^ Allele |  | European Americans | | African Americans | | Latinos | | Japanese Americans | | Native Hawaiians | | Pooled | |
| --- | --- | --- | --- | --- | --- | --- | --- | --- | --- | --- | --- | --- | --- | --- |
|  |  |  | Het | Hom | Het | Hom | Het | Hom | Het | Hom | Het | Hom | Het | Hom |
| rs10923931 | T | OR | 0.79 | 1.03 | 1.24 | 1.05 | 1.20 | 1.12 | 0.98 | NA | 0.76 | 0.64 | 1.10 | 1.00 |
|  |  | 95%CI | 0.59-1.07 | 0.37-2.91 | 1.04-1.48 | 0.78-1.40 | 1.02-1.41 | 0.59-2.11 | 0.68-1.41 |  | 0.49-1.16 | 0.05-8.45 | 1.00-1.22 | 0.78-1.29 |
| rs7578597 | T | OR | 1.94 | 2.64 | 1.06 | 1.09 | 0.52 | 0.60 | 1.05 | 1.16 | NA | NA | 1.00 | 1.16 |
|  |  | 95%CI | 0.58-6.53 | 0.82-8.58 | 0.72-1.58 | 0.75-1.58 | 0.16-1.66 | 0.19-1.91 | 0.06-17.68 | 0.07-18.67 |  |  | 0.72-1.40 | 0.83-1.61 |
| rs1801282 | C | OR | 4.84 | 5.61 | 0.79 | 1.60 | 0.86 | 0.90 | 0.50 | 0.54 | NA | NA | 1.20 | 1.36 |
|  |  | 95%CI | 0.60-38.97 | 0.71-44.57 | 0.06-10.76 | 0.12-21.14 | 0.46-1.59 | 0.49-1.65 | 0.10-2.44 | 0.11-2.63 |  |  | 0.74-1.96 | 0.92-1.20 |
| rs4607103 | C | OR | 1.32 | 1.38 | 1.12 | 1.12 | 1.03 | 0.99 | 1.03 | 1.09 | 0.78 | 0.83 | 1.03 | 1.05 |
|  |  | 95%CI | 0.80-2.19 | 0.84-2.25 | 0.81-1.54 | 0.81-1.54 | 0.82-1.28 | 0.79-1.24 | 0.83-1.28 | 0.87-1.36 | 0.51-1.19 | 0.55-1.26 | 0.91-1.18 | 0.92-1.20 |
| rs4402960 | T | OR | 0.93 | 1.17 | 1.25 | 1.29 | 1.08 | 1.07 | 1.26 | 1.49 | 1.16 | 1.39 | 1.15 | 1.26 |
|  |  | 95%CI | 0.72-1.20 | 0.77-1.77 | 1.01-1.53 | 1.02-1.64 | 0.94-1.23 | 0.85-1.36 | 1.08-1.47 | 1.16-1.92 | 0.92-1.47 | 0.93-2.06 | 1.06-1.24 | 1.11-1.42 |
| rs10010131 | G | OR | 1.44 | 1.5 | 0.98 | 0.9 | 1.05 | 1.24 | NA | NA | 0.79 | 1.11 | 1.05 | 1.19 |
|  |  | 95%CI | 1.01-2.07 | 1.04-2.16 | 0.75-1.28 | 0.69-1.19 | 0.82-1.34 | 0.98-1.58 |  |  | 0.41-1.52 | 0.59-2.09 | 0.90-1.22 | 1.02-1.39 |
| rs7754840 | C | OR | 1.2 | 1.66 | 0.88 | 1.03 | 1.11 | 1.16 | 1.33 | 1.91 | 1.56 | 1.98 | 1.17 | 1.44 |
|  |  | 95%CI | 0.93-1.55 | 1.11-2.48 | 0.70-1.10 | 0.81-1.31 | 0.97-1.26 | 0.93-1.43 | 1.12-1.56 | 1.55-2.35 | 1.15-2.12 | 1.44-2.74 | 1.07-1.27 | 1.30-1.61 |
| rs864745 | T | OR | 0.99 | 0.97 | 1.29 | 1.43 | 1.18 | 1.62 | 0.91 | 1.16 | 1.06 | 1.21 | 1.11 | 1.38 |
|  |  | 95%CI | 0.74-1.34 | 0.69-1.36 | 0.92-1.82 | 1.03-2.00 | 0.97-1.43 | 1.33-1.98 | 0.63-1.30 | 0.82-1.65 | 0.63-1.78 | 0.73-2.01 | 0.98-1.27 | 1.21-1.57 |
| rs13266634 | C | OR | 1.08 | 1.47 | 1.08 | 1.33 | 1.07 | 1.2 | 1.11 | 1.36 | 1.33 | 1.18 | 1.12 | 1.29 |
|  |  | 95%CI | 0.68-1.72 | 0.93-2.32 | 0.44-2.69 | 0.54-3.25 | 0.81-1.42 | 0.91-1.59 | 0.89-1.38 | 1.09-1.70 | 0.95-1.86 | 0.84-1.66 | 0.97-1.29 | 1.12-1.49 |
| rs2383208 | A | OR | 1.65 | 2.13 | 1.15 | 1.31 | 1.3 | 1.47 | 1.26 | 1.58 | 1.14 | 1.11 | 1.26 | 1.46 |
|  |  | 95%CI | 0.78-3.49 | 1.03-4.40 | 0.71-1.86 | 0.82-2.09 | 0.80-2.11 | 0.91-2.35 | 1.03-1.54 | 1.28-1.96 | 0.71-1.83 | 0.70-1.77 | 1.08-1.48 | 1.25-1.70 |
| rs1111875 | C | OR | 1.07 | 0.91 | 1.17 | 1.25 | 0.98 | 1.04 | 1.25 | 1.41 | 0.96 | 0.84 | 1.11 | 1.14 |
|  |  | 95%CI | 0.75-1.52 | 0.63-1.31 | 0.80-1.70 | 0.87-1.81 | 0.81-1.19 | 0.86-1.27 | 1.07-1.45 | 1.08-1.83 | 0.76-1.21 | 0.54-1.30 | 1.00-1.22 | 1.02-1.27 |
| rs7903146 | T | OR | 1.55 | 2.43 | 1.33 | 1.73 | 1.3 | 1.76 | 1.79 | 2.11 | 1.1 | 1.36 | 1.36 | 1.84 |
|  |  | 95%CI | 1.20-2.00 | 1.61-3.67 | 1.11-1.58 | 1.27-2.36 | 1.14-1.48 | 1.37-2.27 | 1.39-2.29 | 0.60-7.40 | 0.85-1.43 | 0.67-2.74 | 1.25-1.48 | 1.56-2.18 |
| rs12779790 | G | OR | 1.06 | 0.9 | 0.97 | 2.07 | 1.19 | 1.4 | 0.98 | 1.15 | 1.18 | 1.29 | 1.07 | 1.33 |
|  |  | 95%CI | 0.81-1.39 | 0.44-1.86 | 0.80-1.17 | 1.16-3.72 | 1.04-1.36 | 0.98-2.01 | 0.83-1.15 | 0.74-1.77 | 0.92-1.51 | 0.74-2.25 | 0.99-1.17 | 1.07-1.66 |
| rs2237895^c^ | C | OR | 1.11 | 0.9 | 1.01 | 1.18 | 1.22 | 1.3 | 1.09 | 1.27 | 1.12 | 1.39 | 1.11 | 1.22 |
|  |  | 95%CI | 0.85-1.45 | 0.63-1.29 | 0.84-1.22 | 0.78-1.78 | 1.05-1.42 | 1.06-1.60 | 0.91-1.30 | 0.97-1.66 | 0.87-1.44 | 0.94-2.04 | 1.02-1.21 | 1.08-1.39 |
| rs2237897^c^ | C | OR | 1.89 | 1.52 | 1.79 | 1.97 | 1.38 | 1.65 | 1.19 | 1.55 | 1.05 | 1.12 | 1.28 | 1.52 |
|  |  | 95%CI | 0.28-12.67 | 0.23-9.85 | 0.44-7.19 | 0.50-7.81 | 1.01-1.89 | 1.20-2.27 | 0.92-1.54 | 1.17-2.05 | 0.60-1.83 | 0.65-1.95 | 1.07-1.53 | 1.27-1.83 |
| rs5219 | T | OR | 1.11 | 1.64 | 1.06 | 0.8 | 1.1 | 1.2 | 1.38 | 1.49 | 0.94 | 1.12 | 1.15 | 1.31 |
|  |  | 95%CI | 0.86-1.44 | 1.15-2.36 | 0.85-1.34 | 0.32-1.96 | 0.96-1.26 | 0.99-1.45 | 1.18-1.62 | 1.18-1.87 | 0.74-1.19 | 0.80-1.57 | 1.06-1.24 | 1.16-1.48 |
| rs7961581 | C | OR | 1.15 | 0.94 | 0.93 | 0.81 | 1.01 | 1.15 | 0.93 | 1.24 | 1.09 | 1.31 | 0.99 | 1.07 |
|  |  |  | 0.89-1.48 | 0.61-1.44 | 0.78-1.12 | 0.55-1.19 | 0.88-1.15 | 0.84-1.56 | 0.80-1.09 | 0.86-1.78 | 0.86-1.38 | 0.86-1.98 | 0.92-1.07 | 0.91-1.26 |
| rs8050136 | A | OR | 0.98 | 0.75 | 0.97 | 1.16 | 1.07 | 0.96 | 1.02 | 1.14 | 1.01 | 1.02 | 1.03 | 1.04 |
|  |  | 95%CI | 0.75-1.28 | 0.52-1.09 | 0.80-1.18 | 0.91-1.47 | 0.93-1.21 | 0.76-1.22 | 0.87-1.19 | 0.80-1.63 | 0.79-1.28 | 0.64-1.63 | 0.95-1.11 | 0.91-1.18 |
| rs4430796 | G | OR | 0.83 | 0.93 | 1.26 | 1.29 | 0.99 | 0.92 | 1.21 | 1.38 | 1.01 | 1.32 | 1.06 | 1.11 |
|  |  | 95%CI | 0.62-1.11 | 0.67-1.29 | 0.95-1.66 | 0.98-1.71 | 0.86-1.13 | 0.77-1.10 | 1.03-1.41 | 1.10-1.73 | 0.80-1.27 | 0.90-1.96 | 0.98-1.16 | 0.99-1.23 |

Het = heterozygous for risk allele; Hom = homozygous for risk allele; NA = small number of individuals in a cell and can not be estimated

^a^ORs adjusted for age (quartiles), BMI (quartiles), sex, and ethnicity (pooled analysis)

^b^NCBI build 36 (forward strand)

^c^rs2237895 and rs2237897 adjusted for one another
