# Supplementary material for: Consistent Association of Type 2 Diabetes Risk Variants Found in Europeans in Diverse Racial and Ethnic Groups
Source: PLoS Genet. 2010 Aug 26;6(8):e1001078. doi: 10.1371/journal.pgen.1001078 (PMC2928808; doi:10.1371/journal.pgen.1001078)
Supplement: Table S3 — Effects of European ancestry adjustment in African Americans. (0.07 MB DOC) [file pgen.1001078.s003.doc]

**Table S3: Effects of European ancestry adjustment in African Americans.**

|  |  |  |  |  |  | African American Males with  % European ancestry estimates  (336 cases, 397 controls) | |
| --- | --- | --- | --- | --- | --- | --- | --- |
| SNP | Chr | Nearest  Gene | Risk  Allele^a^ | Risk Allele  Frequency,  European  Americans | Risk Allele Frequency, African American | Unadjusted  OR(95% CI)^b^  per allele | Adjusted  OR(95% CI)^c^  per allele |
| rs10923931 | 1 | *NOTCH2* | T | 0.12 | 0.29 | 0.94(0.74-1.19) | 0.93(0.73-1.18) |
| rs7578597 | 2 | *THADA* | T | 0.90 | 0.75 | 1.15(0.89-1.49) | 1.16(0.90-1.51) |
| rs1801282 | 3 | *PPARG* | C | 0.89 | 0.97 | 2.42(1.18-4.98) | 2.39(1.16-4.92) |
| rs4607103 | 3 | *ADAMTS9* | C | 0.73 | 0.70 | 1.03(0.81-1.31) | 1.04(0.81-1.32) |
| rs4402960 | 3 | *IGF2BP2* | C | 0.31 | 0.49 | 0.82(0.67-1.02) | 0.81(0.66-1.01) |
| rs10010131 | 4 | *WFS1* | G | 0.59 | 0.66 | 1.01(0.80-1.27) | 1.01(0.80-1.27) |
| rs7754840 | 6 | *CDKAL1* | C | 0.29 | 0.55 | 1.10(0.88-1.37) | 1.09(0.87-1.36) |
| rs864745 | 7 | *JAZF1* | T | 0.51 | 0.73 | 1.26(0.99-1.61) | 1.26(0.98-1.61) |
| rs13266634 | 8 | *SLC30A8* | C | 0.68 | 0.89 | 1.37(0.95-1.97) | 1.35(0.92-1.96) |
| rs2383208 | 9 | *CDKN2B* | T | 0.81 | 0.81 | 1.05(0.79-1.39) | 1.04(0.78-1.38) |
| rs1111875 | 10 | *HHEX* | C | 0.61 | 0.74 | 0.96(0.74-1.25) | 0.95(0.73-1.23) |
| rs7903146 | 10 | *TCF7L2* | T | 0.27 | 0.28 | 1.39(1.08-1.79) | 1.39(1.08-1.79) |
| rs12779790 | 10 | *CDC123* | G | 0.17 | 0.14 | 1.06(0.78-1.43) | 1.06(0.78-1.43) |
| rs2237895^d^ | 11 | *KCNQ1* | C | 0.42 | 0.20 | 0.96(0.73-1.26) | 0.96(0.73-1.27) |
| rs2237897^d^ | 11 | *KCNQ1* | C | 0.95 | 0.92 | 1.58(0.99-2.54) | 1.59(0.99-2.55) |
| rs5219 | 11 | *KCNJ11* | T | 0.35 | 0.09 | 1.32(0.90-1.92) | 1.38(0.93-2.04) |
| rs7961581 | 12 | *TSPAN8* | C | 0.29 | 0.23 | 0.87(0.67-1.12) | 0.88(0.68-1.13) |
| rs8050136 | 16 | *FTO* | A | 0.41 | 0.43 | 1.12(0.90-1.39) | 1.12(0.90-1.39) |
| rs4430796 | 17 | *HNF1B* | G | 0.50 | 0.65 | 1.13(0.90-1.43) | 1.12(0.89-1.42) |

^a^NCBI build 36 (forward strand)

^b^Adjusted for age (quartiles), and BMI (quartiles)

^c^Adjusted for age (quartiles), BMI (quartiles), and % European ancestry (estimated using ancestry informative markers as previously designed [16,30,31])

^d^rs2237895 and rs2237897 adjusted for one another
