# Supplementary material for: Consistent Association of Type 2 Diabetes Risk Variants Found in Europeans in Diverse Racial and Ethnic Groups
Source: PLoS Genet. 2010 Aug 26;6(8):e1001078. doi: 10.1371/journal.pgen.1001078 (PMC2928808; doi:10.1371/journal.pgen.1001078)
Supplement: Table S4 — Effects of adjustment for education. (0.12 MB DOC) [file pgen.1001078.s004.doc]

**Table S4: Effects of adjustment for education.**

|  |  |  | European Americans  529 cases  1,000 controls^a^ | | African Americans  1,067 case  1,464 controls^a^ | | Latinos  2,190 case  2,157 controls^a^ | | Japanese Americans  1,727 case  1,756 controls^a^ | | Native Hawaiians  573 cases  973 controls^a^ | | Pooled  6,110 cases  7,799 controls^a^ | |
| --- | --- | --- | --- | --- | --- | --- | --- | --- | --- | --- | --- | --- | --- | --- |
| SNP | Risk Allele^b^ |  | Unadj^c^ | Adj^d^ | Unadj^c^ | Adj^d^ | Unadj^c^ | Adj^d^ | Unadj^c^ | Adj^d^ | Unadj^c^ | Adj^d^ | Unadj^c^ | Adj^d^ |
| rs10923931 | T | OR | 0.83 | 0.85 | 1.1 | 1.09 | 1.15 | 1.15 | 1 | 1 | 0.76 | 0.76 | 1.06 | 1.05 |
|  |  | 95%CI | 0.63-1.09 | 0.65-1.11 | 0.96-1.24 | 0.96-1.24 | 0.99-1.34 | 0.99-1.33 | 0.70-1.44 | 0.69-1.43 | 0.50-1.14 | 0.50-1.14 | 0.97-1.15 | 0.97-1.15 |
| rs7578597 | T | OR | 1.41 | 1.34 | 1.04 | 1.04 | 1.11 | 1.11 | 1.1 | 1.11 | 1.66 | 1.63 | 1.13 | 1.12 |
|  |  | 95%CI | 1.05-1.89 | 1.00-1.81 | 0.91-1.20 | 0.91-1.20 | 0.92-1.34 | 0.92-1.34 | 0.68-1.78 | 0.69-1.80 | 1.02-2.71 | 1.00-2.66 | 1.02-1.25 | 1.02-1.24 |
| rs1801282 | C | OR | 1.25 | 1.24 | 1.92 | 1.88 | 1.02 | 1.02 | 1.05 | 1.05 | 1.16 | 1.15 | 1.13 | 1.13 |
|  |  | 95%CI | 0.94-1.67 | 0.93-1.65 | 1.28-2.88 | 1.25-2.82 | 0.88-1.18 | 0.88-1.19 | 0.81-1.35 | 0.82-1.36 | 0.85-1.57 | 0.85-1.56 | 1.01-1.25 | 1.02-1.25 |
| rs4607103 | C | OR | 1.11 | 1.12 | 1.04 | 1.03 | 0.98 | 0.98 | 1.05 | 1.06 | 0.99 | 0.98 | 1.02 | 1.02 |
|  |  | 95%CI | 0.91-1.34 | 0.92-1.36 | 0.91-1.18 | 0.91-1.18 | 0.89-1.08 | 0.89-1.08 | 0.95-1.17 | 0.95-1.17 | 0.83-1.17 | 0.83-1.17 | 0.97-1.08 | 0.97-1.08 |
| rs4402960 | T | OR | 1.01 | 0.99 | 1.14 | 1.14 | 1.06 | 1.06 | 1.24 | 1.24 | 1.18 | 1.19 | 1.13 | 1.13 |
|  |  | 95%CI | 0.84-1.22 | 0.82-1.19 | 1.01-1.28 | 1.01-1.28 | 0.96-1.17 | 0.96-1.17 | 1.11-1.38 | 1.11-1.38 | 0.99-1.40 | 1.00-1.41 | 1.07-1.20 | 1.07-1.20 |
| rs10010131 | G | OR | 1.18 | 1.17 | 0.94 | 0.94 | 1.14 | 1.14 | 1.45 | 1.43 | 1.25 | 1.25 | 1.11 | 1.1 |
|  |  | 95%CI | 0.99-1.40 | 0.98-1.39 | 0.83-1.07 | 0.83-1.06 | 1.03-1.26 | 1.03-1.26 | 0.98-2.13 | 0.97-2.11 | 1.02-1.54 | 1.01-1.53 | 1.04-1.18 | 1.03-1.18 |
| rs7754840 | C | OR | 1.24 | 1.21 | 1.03 | 1.02 | 1.07 | 1.07 | 1.38 | 1.39 | 1.38 | 1.37 | 1.19 | 1.19 |
|  |  | 95%CI | 1.04-1.49 | 1.01-1.45 | 0.91-1.16 | 0.91-1.15 | 0.97-1.18 | 0.97-1.17 | 1.25-1.53 | 1.25-1.54 | 1.18-1.62 | 1.17-1.61 | 1.13-1.26 | 1.13-1.25 |
| rs864745 | T | OR | 0.98 | 0.99 | 1.16 | 1.16 | 1.3 | 1.29 | 1.19 | 1.19 | 1.12 | 1.11 | 1.19 | 1.19 |
|  |  | 95%CI | 0.83-1.16 | 0.84-1.17 | 1.01-1.32 | 1.01-1.33 | 1.18-1.42 | 1.18-1.42 | 1.05-1.35 | 1.05-1.35 | 0.93-1.35 | 0.92-1.34 | 1.13-1.26 | 1.12-1.26 |
| rs13266634 | C | OR | 1.28 | 1.28 | 1.22 | 1.21 | 1.11 | 1.11 | 1.18 | 1.18 | 1.03 | 1.04 | 1.15 | 1.14 |
|  |  | 95%CI | 1.06-1.55 | 1.05-1.55 | 1.00-1.49 | 0.99-1.48 | 1.00-1.24 | 1.00-1.23 | 1.06-1.32 | 1.06-1.31 | 0.88-1.21 | 0.88-1.22 | 1.08-1.22 | 1.08-1.21 |
| rs2383208 | A | OR | 1.33 | 1.37 | 1.14 | 1.14 | 1.15 | 1.15 | 1.25 | 1.25 | 1.02 | 1.02 | 1.18 | 1.18 |
|  |  | 95%CI | 1.06-1.67 | 1.09-1.73 | 0.98-1.33 | 0.98-1.33 | 1.01-1.31 | 1.01-1.31 | 1.13-1.39 | 1.13-1.39 | 0.85-1.22 | 0.86-1.23 | 1.11-1.26 | 1.11-1.26 |
| rs1111875 | C | OR | 0.92 | 0.93 | 1.11 | 1.1 | 1.03 | 1.03 | 1.21 | 1.21 | 0.94 | 0.94 | 1.07 | 1.07 |
|  |  | 95%CI | 0.78-1.10 | 0.78-1.11 | 0.96-1.27 | 0.96-1.27 | 0.94-1.13 | 0.94-1.13 | 1.08-1.36 | 1.08-1.36 | 0.78-1.12 | 0.79-1.13 | 1.01-1.13 | 1.01-1.13 |
| rs7903146 | T | OR | 1.54 | 1.55 | 1.32 | 1.32 | 1.31 | 1.32 | 1.78 | 1.77 | 1.12 | 1.11 | 1.36 | 1.36 |
|  |  | 95%CI | 1.28-1.85 | 1.29-1.87 | 1.16-1.51 | 1.16-1.51 | 1.19-1.45 | 1.19-1.46 | 1.41-2.25 | 1.40-2.24 | 0.90-1.39 | 0.90-1.39 | 1.27-1.45 | 1.27-1.46 |
| rs12779790 | G | OR | 1.01 | 1.04 | 1.09 | 1.1 | 1.2 | 1.2 | 1.03 | 1.02 | 1.17 | 1.17 | 1.11 | 1.11 |
|  |  | 95%CI | 0.80-1.26 | 0.83-1.30 | 0.92-1.29 | 0.93-1.30 | 1.07-1.35 | 1.07-1.35 | 0.89-1.18 | 0.89-1.17 | 0.96-1.43 | 0.96-1.43 | 1.04-1.19 | 1.04-1.19 |
| rs2237895 | C | OR | 0.98 | 0.98 | 1.05 | 1.06 | 1.15 | 1.14 | 1.12 | 1.11 | 1.16 | 1.16 | 1.11 | 1.11 |
|  |  | 95%CI | 0.82-1.17 | 0.82-1.17 | 0.91-1.22 | 0.91-1.23 | 1.04-1.27 | 1.03-1.26 | 0.98-1.27 | 0.98-1.26 | 0.97-1.39 | 0.97-1.39 | 1.04-1.18 | 1.04-1.17 |
| rs2237897 | C | OR | 0.85 | 0.87 | 1.13 | 1.13 | 1.24 | 1.25 | 1.27 | 1.27 | 1.07 | 1.07 | 1.22 | 1.22 |
|  |  | 95%CI | 0.58-1.24 | 0.59-1.28 | 0.90-1.42 | 0.90-1.42 | 1.10-1.40 | 1.11-1.41 | 1.12-1.45 | 1.12-1.45 | 0.87-1.32 | 0.87-1.32 | 1.13-1.31 | 1.13-1.31 |
| rs5219 | T | OR | 1.25 | 1.26 | 1.02 | 1.03 | 1.1 | 1.1 | 1.26 | 1.27 | 1.03 | 1.03 | 1.15 | 1.15 |
|  |  | 95%CI | 1.05-1.48 | 1.06-1.50 | 0.83-1.25 | 0.84-1.26 | 1.00-1.21 | 1.00-1.20 | 1.14-1.41 | 1.14-1.41 | 0.88-1.21 | 0.88-1.21 | 1.08-1.22 | 1.09-1.22 |
| rs7961581 | C | OR | 1.03 | 1.04 | 0.92 | 0.92 | 1.03 | 1.03 | 1 | 0.99 | 1.12 | 1.12 | 1.01 | 1.01 |
|  |  | 95%CI | 0.86-1.23 | 0.86-1.25 | 0.80-1.06 | 0.80-1.06 | 0.92-1.15 | 0.93-1.15 | 0.88-1.13 | 0.88-1.13 | 0.94-1.34 | 0.94-1.33 | 0.95-1.07 | 0.95-1.07 |
| rs8050136 | A | OR | 0.89 | 0.91 | 1.06 | 1.06 | 1.02 | 1.02 | 1.03 | 1.04 | 1.01 | 1.02 | 1.02 | 1.02 |
|  |  | 95%CI | 0.75-1.06 | 0.76-1.08 | 0.94-1.19 | 0.94-1.19 | 0.92-1.13 | 0.93-1.13 | 0.91-1.17 | 0.91-1.17 | 0.84-1.21 | 0.85-1.22 | 0.96-1.08 | 0.96-1.08 |
| rs4430796 | G | OR | 0.97 | 0.95 | 1.11 | 1.11 | 0.96 | 0.96 | 1.18 | 1.18 | 1.09 | 1.1 | 1.05 | 1.05 |
|  |  | 95%CI | 0.82-1.14 | 0.81-1.13 | 0.98-1.26 | 0.98-1.26 | 0.87-1.04 | 0.87-1.04 | 1.06-1.31 | 1.06-1.32 | 0.92-1.29 | 0.92-1.30 | 1.00-1.11 | 1.00-1.11 |

^a^Missing data (n cases/ n controls): European American (4/6); African American (10/5); Latino (30/27); Japanese (9/5); and Native Hawaiian (3/10)

^b^NCBI build 36 (forward strand)

^c^Adjusted for age (quartiles), BMI (quartiles), sex, and ethnicity (pooled)

^d^Adjusted for age (quartiles), BMI (quartiles), sex, education (categorized as: high school graduate or less; some college or vocational school; and college graduate or graduate or professional school), and ethnicity (pooled)
