# Supplementary material for: Consistent Association of Type 2 Diabetes Risk Variants Found in Europeans in Diverse Racial and Ethnic Groups
Source: PLoS Genet. 2010 Aug 26;6(8):e1001078. doi: 10.1371/journal.pgen.1001078 (PMC2928808; doi:10.1371/journal.pgen.1001078)
Supplement: Table S5 — Associations with risk score among subjects with and without complete genotype data. (0.05 MB DOC) [file pgen.1001078.s005.doc]

**Table S5: Associations with risk score among subjects with and without complete genotype data.a**

|  | European Americans | African Americans | Latinos | Japanese Americans | Native Hawaiians | Pooled | P_het_^b^ |
| --- | --- | --- | --- | --- | --- | --- | --- |
|  | All subjects and all variants. Missing genotype data assigned as mean number of alleles (6,142 cases and 7,403 controls). | | | | | |  |
| N (Cases/Controls) | 533/1,006 | 1,077/1,469 | 2,220/2,184 | 1,736/1,761 | 576/983 | 6,142/7,403 |  |
| OR (95% CI) | 1.11 (1.06-1.17) | 1.09 (1.05-1.12) | 1.12 (1.09-1.14) | 1.20 (1.17-1.24) | 1.10 (1.06-1.15) | 1.13 (1.11-1.15) | 3.8 x 10^-4^ |
| P-Value | 1.2 x 10^-5^ | 3.0 x 10^-6^ | 7.5 x 10^-19^ | 7.0 x 10^-32^ | 1.2 x 10^-5^ | 4.7 x 10^-59^ |  |
|  | All subjects with complete genotype data for all variants (5,522 cases and 6,633 controls). | | | | | |  |
| N (Cases/Controls) | 442/890 | 975/1,298 | 2,023/1,996 | 1,564/1,571 | 518/878 | 5,522/6,633 |  |
| OR (95% CI) | 1.11 (1.06-1.17) | 1.09 (1.05-1.13) | 1.12 (1.09-1.14) | 1.19 (1.15-1.23) | 1.10 (1.06-1.16) | 1.13 (1.11-1.15) | 5.7 x 10^-3^ |
| P-Value | 4.1 x 10^-5^ | 2.3 x 10^-6^ | 2.0 x 10^-17^ | 6.0 x 10^-27^ | 2.0 x 10^-5^ | 2.7 x 10^-53^ |  |
|  | All subjects and all variants with P<0.10 in ethnic-pooled analysis. Missing genotype data assigned as mean number of alleles (6,142 cases 7,403 controls). | | | | | |  |
| N (Cases/Controls) | 533/1,006 | 1,077/1,469 | 2,220/2,184 | 1,736/1,761 | 576/983 | 6,142/7,403 |  |
| OR (95% CI) | 1.13 (1.07-1.18) | 1.10 (1.06-1.15) | 1.13 (1.10-1.16) | 1.23 (1.19-1.28) | 1.12 (1.07-1.17) | 1.15 (1.13-1.17) | 2.4 x 10^-4^ |
| P-Value | 5.1 x 10^-6^ | 6.4 x 10^-7^ | 4.9 x 10^-20^ | 3.5 x 10^-35^ | 4.5 x 10^-6^ | 8.1 x 10^-65^ |  |
|  | All subjects with complete genotype data using only variants with P<0.10 in ethnic-pooled analysis (5,572 cases 6,682 controls). | | | | | |  |
| N (Cases/Controls) | 450/897 | 953/1,311 | 2,039/2,010 | 1,571/1,578 | 521/886 | 5,572/6,682 |  |
| OR (95% CI) | 1.12 (1.07-1.19) | 1.11 (1.07-1.15) | 1.13 (1.10-1.16) | 1.23 (1.18-1.27) | 1.12 (1.06-1.17) | 1.15 (1.13-1.17) | 2.1 x 10^-3^ |
| P-Value | 2.7 x 10^-5^ | 4.6 x 10^-7^ | 4.9 x 10^-18^ | 1.5 x 10^-30^ | 1.1 x 10^-5^ | 3.5 x 10^-58^ |  |

^a^Odds ratios adjusted for age (quartiles), BMI (quartiles), sex, and ethnicity (in pooled analysis)

^b^P_het_ = P value for heterogeneity of allelic effects across ethnic groups (4 df test)
