# Supplementary material for: Consistent Association of Type 2 Diabetes Risk Variants Found in Europeans in Diverse Racial and Ethnic Groups
Source: PLoS Genet. 2010 Aug 26;6(8):e1001078. doi: 10.1371/journal.pgen.1001078 (PMC2928808; doi:10.1371/journal.pgen.1001078)
Supplement: Table S6 — Genotyping efficiency: genotype call rates, Hardy-Weinberg Equilibrium testing. (0.08 MB DOC) [file pgen.1001078.s006.doc]

**Table S6: Genotyping efficiency: genotype call rates, Hardy-Weinberg Equilibrium testing.**

| SNP |  | European Americans  533 cases  1,006 controls | African Americans  1,077 cases  1,469 controls | Latinos  2,220 cases  2,184 controls | Japanese Americans  1,736 cases  1,761 controls | Native Hawaiians  576 cases  983 controls |
| --- | --- | --- | --- | --- | --- | --- |
| rs10923931 | Call Rate (Ca/Co) | 100%/99.3% | 98.8%/98.8% | 99.7%/99.6% | 99.8%/99.8% | 99.5%/99.5% |
|  | HWE P-value (Ca/Co) | 0.92/0.72 | 0.40/0.034 | 0.64/0.83 | 0.87/0.40 | 0.59/0.78 |
| rs7578597 | Call Rate (Ca/Co) | 99,2%/100% | 99.7%/99.5% | 99.8%/99.5% | 99.8%/99.4% | 99.8%/99.7% |
|  | HWE P-value (Ca/Co) | 0.47/0.15 | 0.39/0.55 | 0.58/0.18 | 0.048/0.066 | 0.18/0.27 |
| rs1801282 | Call Rate (Ca/Co) | 100%/99.9% | 99.7%/99.5% | 99.5%/99.5% | 99.9%/99.6% | 99.1%/99.6% |
|  | HWE P-value (Ca/Co) | 0.084/0.20 | 0.20/0.70 | 0.41/0.99 | 0.43/0.99 | 0.084/0.091 |
| rs4607103 | Call Rate (Ca/Co) | 99.2%/99.7% | 99.7%/99.5% | 99.5%/99.5% | 99.8%/99.3% | 99.7%/99.4% |
|  | HWE P-value (Ca/Co) | 0.82/0.68 | 0.40/0.48 | 0.10/0.44 | 0.71/0.72 | 0.14/0.92 |
| rs4402960 | Call Rate (Ca/Co) | 99.1%/98.8% | 99.4%/98.9% | 99.7%/99.4% | 99.4%/99.0% | 99.3%/98.6% |
|  | HWE P-value (Ca/Co) | 0.80/0.20 | 0.16/0.79 | 0.35/0.10 | 0.33/0.39 | 0.23/0.39 |
| rs10010131 | Call Rate (Ca/Co) | 98.9%/99.5% | 99.8%/99.3% | 99.7%/99.7% | 99.8%/99.7% | 99.8%/99.8% |
|  | HWE P-value (Ca/Co) | 0.87/0.41 | 0.60/0.45 | 0.61/0.97 | 0.013/0.40 | 0.28/0.71 |
| rs7754840 | Call Rate (Ca/Co) | 97.9%/97.8% | 99.7%/99.4% | 99.8%/99.4% | 96.9%/98.2% | 98.1%/98.3% |
|  | HWE P-value (Ca/Co) | 0.30/0.24 | 0.15/0.99 | 0.62/0.31 | 0.074/0.30 | 0.60/0.15 |
| rs864745 | Call Rate (Ca/Co) | 97.6%/99.1% | 98.4%/99.0% | 98.5%/98.9% | 98.6%/98.4% | 98.4%/97.5% |
|  | HWE P-value (Ca/Co) | 0.89/0.98 | 0.62/0.024 | 0.88/0.13 | 0.39/0.24 | 0.59/0.71 |
| rs13266634 | Call Rate (Ca/Co) | 100%/99.1% | 99.8%/99.5% | 99.8%/99.5% | 99.7%/99.5% | 99.8%/98.8% |
|  | HWE P-value (Ca/Co) | 0.97/0.045 | 0.88/0.87 | 0.64/0.61 | 0.66/0.37 | 0.49/0.082 |
| rs2383208 | Call Rate (Ca/Co) | 96.2%/95.2% | 99.4%/98.5% | 99.6%/99.4% | 98.0%/97.1% | 97.9%/98.3% |
|  | HWE P-value (Ca/Co) | 0.96/0.76 | 0.81/0.97 | 0.45/0.64 | 0.58/0.53 | 0.54/0.67 |
| rs1111875 | Call Rate (Ca/Co) | 98.9%/99.2% | 99.0%/98.6% | 99.0%/99.7% | 99.4%/99.5% | 99.7%/99.5% |
|  | HWE P-value (Ca/Co) | 0.13/0.19 | 0.48/0.94 | 0.66/0.79 | 0.51/0.74 | 0.94/0.90 |
| rs7903146 | Call Rate (Ca/Co) | 96.6%/99.3% | 99.3%/98.4% | 99.7%/98.9% | 99.2%/98.8% | 98.8%/99.0% |
|  | HWE P-value (Ca/Co) | 0.44/0.62 | 0.19/0.28 | 0.10/0.19 | 0.87/0.25 | 0.16/0.14 |
| rs12779790 | Call Rate (Ca/Co) | 96.8%/98.3% | 99.0%/99.3% | 98.8%/99.3% | 97.7%/97.2% | 99.0%/98.8% |
|  | HWE P-value (Ca/Co) | 0.87/0.87 | 0.094/0.25 | 0.73/0.75 | 0.19/0.46 | 0.31/0.19 |
| rs2237895 | Call Rate (Ca/Co) | 99.2%/99.4% | 99.4%/98.8% | 99.7%/99.8% | 98.4%/99.4% | 98.1%/99.4% |
|  | HWE P-value (Ca/Co) | 0.86/0.67 | 0.18/0.80 | 0.26/0.76 | 0.23/0.71 | 0.19/0.86 |
| rs2237897 | Call Rate (Ca/Co) | 99.4%/98.8% | 99.6%/99.0% | 99.3%/99.4% | 98.4%/98.8% | 98.4%/97.5% |
|  | HWE P-value (Ca/Co) | 0.81/0.050 | 0.26/0.91 | 0.73/0.26 | 0.67/0.62 | 0.56/0.75 |
| rs5219 | Call Rate (Ca/Co) | 98.3%/98.2% | 99.1%/99.0% | 98.6%/99.0% | 98.6%/98.6% | 99.7%/99.0% |
|  | HWE P-value (Ca/Co) | 0.062/0.84 | 0.79/0.27 | 0.99/0.81 | 0.27/0.65 | 0.19/0.70 |
| rs7961581 | Call Rate (Ca/Co) | 98.5%/98.5% | 99.1%/99.3% | 99.5%/99.5% | 98.7%/98.8% | 99.7%/99.2% |
|  | HWE P-value (Ca/Co) | 0.30/0.29 | 0.58/0.35 | 0.71/0.89 | 0.35/0.24 | 0.91/0.86 |
| rs8050136 | Call Rate (Ca/Co) | 97.9%/99.4% | 99.7%/99.4% | 99.7%/99.6% | 99.3%/99.2% | 98.8%/98.7% |
|  | HWE P-value (Ca/Co) | 0.32/0.99 | 0.12/0.59 | 0.92/0.24 | 0.30/0.38 | 0.60/0.16 |
| rs4430796 | Call Rate (Ca/Co) | 97.7%/99.1% | 99.5%/98.4% | 99.3%/98.9% | 99.5%/99.1% | 99.8%/99.1% |
|  | HWE P-value (Ca/Co) | 0.27/0.64 | 0.53/0.95 | 0.13/0.026 | 0.53/0.66 | 0.93/0.084 |
